# Longevity-associated mitochondrial DNA 5178 C/A polymorphism modulates the effects of coffee consumption on erythrocytic parameters in Japanese men: an exploratory cross-sectional analysis

**DOI:** 10.1186/1880-6805-33-37

**Published:** 2014-12-20

**Authors:** Akatsuki Kokaze, Mamoru Ishikawa, Naomi Matsunaga, Kanae Karita, Masao Yoshida, Tadahiro Ohtsu, Hirotaka Ochiai, Takako Shirasawa, Hinako Nanri, Nobuyuki Saga, Iichiro Ohtsu, Hiromi Hoshino, Yutaka Takashima

**Affiliations:** Department of Public Health, Showa University School of Medicine, 1-5-8 Hatanodai, Shinagawa-ku, Tokyo, 142-8555 Japan; Department of Public Health, Kyorin University School of Medicine, 6-20-2 Shinkawa, Mitaka-shi, Tokyo, 181-8611 Japan; Mito Red Cross Hospital, 3-12-48 Sannomaru, Mito-shi, Ibaraki, 310-0011 Japan

**Keywords:** Anemia, Coffee consumption, Hemoglobin, Mitochondrial DNA polymorphism, Personalized preventive medicine, Red blood cell

## Abstract

**Background:**

Mitochondrial DNA 5178 cytosine/adenine (Mt5178 C/A) polymorphism reportedly modulates the effects of coffee consumption on the risk of hypertension, dyslipidemia and abnormal glucose tolerance. The objective of this analysis was to investigate whether Mt5178 C/A polymorphism modifies the effects of coffee consumption on erythrocytic parameters in male Japanese health check-up examinees.

**Methods:**

A total of 436 men (mean age ± standard deviation, 54.1 ± 7.8 years) were selected from among individuals visiting the hospital for regular medical check-ups. After Mt5178 C/A genotyping, an exploratory cross-sectional analysis assessing the joint effects of Mt5178 C/A polymorphism and coffee consumption on red blood cell counts, hematocrit and hemoglobin was conducted.

**Results:**

For Mt5178C genotypic men, after adjustment for age, body mass index, alcohol consumption, habitual smoking and green tea consumption, coffee consumption significantly decreased red blood cell counts (*P* for trend *=* 0.022) and hemoglobin (*P* for trend = 0.035). The risk of anemia, defined as hemoglobin of <14 g/dL, after the aforementioned adjustment, appeared to depend on coffee consumption (*P* for trend = 0.078), and the adjusted odds ratio for anemia was significantly higher in men who consumed ≥4 cups of coffee per day than in those who consumed <1 cup per day (odds ratio = 3.771, 95% confidence interval: 1.088 to 13.06, *P* = 0.036). For Mt5178A genotypic men, coffee consumption possibly reduced the risk of anemia (*P* for trend = 0.049). However, after the aforementioned adjustment, the statistical significance disappeared (*P* for trend = 0.137).

**Conclusions:**

This exploratory cross-sectional analysis suggests that Mt5178 C/A polymorphism modulates the effects of coffee consumption on erythrocytic parameters and the risk of anemia in male Japanese health check-up examinees.

## Background

Studying culturally diverse populations increases the potential of revealing gene-environment interactions [1]. Personalized medicine utilizes gene-environment interactions for the prevention, diagnosis and treatment of diseases [2]. From the viewpoint of prevention, information on gene-environment interactions is necessary to determine the individualized optimum for maximizing the risk reduction and minimizing the risk increase of behavior for lifestyle-related diseases.

Mitochondrial DNA sequences are utilized in studies of the history and evolution of human populations [3]. In physiological anthropology, Nishimura *et al*. reported the interaction between mitochondrial DNA haplogroups and seasonal cold acclimatization [4]. Mitochondrial DNA cytosine/adenine (Mt5178 C/A) polymorphism, also known as NADH dehydrogenase subunit-2 237 leucine/methionine (ND2-237 Leu/Met) polymorphism, is associated with longevity in the Japanese population [5]. The frequency of the Mt5178A genotype is significantly higher in Japanese centenarians than in the general population [5]. Japanese individuals with Mt5178A are more resistant to hypertension [6], diabetes [7], myocardial infarction [8, 9] and cerebrovascular disorders [10] than those with Mt5178C. Moreover, Mt5178 C/A polymorphism interacts with several lifestyle habits, namely habitual smoking [11–14], alcohol consumption [6, 14, 15], coffee consumption [16–18] and green tea consumption [19], on the risk of lifestyle-related diseases. Mt5178 C/A polymorphism influences the effects of coffee consumption on the risk of hypertension [16], hyper-low-density lipoprotein (LDL) cholesterolemia [17] and the clustering of cardiovascular risk factors [18].

Although coffee intake appears to be a beneficial lifestyle behavior for health [20, 21], several researchers have reported that coffee consumption is associated with anemia [22–24], and this has been the focus of bioarchaeological studies [25, 26]. Mitochondria play many crucial roles in hematopoietic cell homeostasis [27]. We have previously reported the joint effects of Mt5178 C/A polymorphism and cigarette smoking on erythrocytic parameters [11]. However, there have been no studies on the gene-environment interaction between Mt5178 C/A polymorphism and coffee consumption on erythrocytic parameters.

The objective of this study was to investigate whether there is a combined effect of longevity-associated Mt5178 C/A polymorphism and coffee consumption on erythrocytic parameters in male Japanese health check-up examinees.

## Methods

### Participants

Participants were recruited from among individuals visiting the Mito Red Cross Hospital for regular medical check-ups between August 1999 and August 2000. This study was conducted in accordance with the Declaration of Helsinki and was approved by the Ethics Committee of the Kyorin University School of Medicine. Written informed consent was obtained from 602 volunteers before participation. Because the number of women was insufficient for classification into groups based on Mt5178 C/A genotype and coffee consumption, female health check-up examinees were excluded. Male health check-up examinees with unclear data were also excluded. Therefore, the subjects of this analysis comprised 436 Japanese men (mean age ± SD, 54.1 ± 7.8 years).

### Clinical characteristics of participants

Hematological parameters, determined using an automated counter (Sysmex SE9000; Sysmex, Kobe, Japan), were obtained from the results of regular medical check-ups. Anemia was defined as hemoglobin of <14 g/dL [28]. Body mass index (BMI) was defined as the ratio of the individual’s weight (kg) to the square of their height (m). A survey of coffee intake, habitual smoking, alcohol consumption and green tea intake was performed using a questionnaire. As in previous reports [17, 18], coffee consumption was categorized based on the number of cups of coffee per day (<1 cup per day, 1 to 3 cups per day, ≥4 cups per day). Habitual smoking was classified as non- or ex-smokers and current smokers. Alcohol consumption was classified based on drinking frequency (daily drinkers; occasional drinkers, which included those who drink several times per week or per month; and non- or ex-drinkers). Green tea consumption was classified based on the number of cups of green tea per day (<1 cup per day, 1 to 4 cups per day, ≥5 cups per day).

### Genotyping

DNA was extracted from white blood cells using a DNA Extractor WB kit (Wako Pure Chemical Industries, Osaka, Japan). Mt5178 C/A polymorphism was detected by polymerase chain reaction (PCR) and digestion with *Alu*I restriction enzyme. The sequence of primers was: forward 5′-CTTAGCATACTCCTCAATTACCC-3′ and reverse 5′-GTGAATTCTTCGATAATGGCCCA-3′. PCR was performed with 50 ng genomic DNA in a buffer containing 0.2 μmol/L of each primer, 1.25 mmol/L deoxyribonucleotide triphosphates, 1.5 mmol/L MgCl_2_ and 1 U of Taq DNA polymerase. After initial denaturation at 94°C for 5 min, PCR was conducted for 40 cycles in the following steps: denaturation at 94°C for 30 s, annealing at 60°C for 60 s and polymerase extension at 72°C for 90 s. After cycling, a final extension at 72°C for 10 min was performed. PCR products were digested with *Alu*I restriction enzyme (Nippon Gene, Tokyo, Japan) at 37°C overnight, and were electrophoresed in 1.5% agarose gels stained with ethidium bromide for visualization under ultraviolet light. The absence of an *Alu*I site was designated as Mt5178A, and the presence of this restriction site was designated as Mt5178C.

### Statistical analyses

Statistical analyses were performed using SAS statistical software version 9.2 for Windows (SAS Institute, Inc., Cary, NC, USA). Multiple logistic regression analysis was used to calculate odds ratios (ORs) for the risk of anemia (hemoglobin <14 g/dL). For multiple logistic regression analysis and analysis of covariance, habitual smoking (non- or ex-smokers = 0, current smokers = 1), alcohol consumption (non- or ex-drinkers = 0, occasional drinkers, including those who drink several times per week or per month = 1, daily drinkers = 2) and green tea consumption (<1 cup per day = 1, 1 to 3 cups per day = 2, ≥5 cups per day = 3) were numerically coded. Differences with *P* values of less than 0.05 were considered to be statistically significant.

## Results

In our study population, the frequency of Mt5178C was 41.0% and that of Mt5178A genotype was 39.0% (Table 1). No significant differences in erythrocytic parameters, namely red blood cell (RBC) count, hematocrit, hemoglobin, mean corpuscular volume, mean corpuscular hemoglobin or mean corpuscular hemoglobin concentration, were observed between the Mt5178C and Mt5178A genotypes. However, white blood cell count was significantly higher in Mt5178A genotypic men than in Mt5178C genotypic men (*P* = 0.026).

**Table 1:** **Hematological parameters of study participants by Mt5178 C/A genotype**

	Mt5178C	Mt5178A	***P***value
	N = 266	N = 170	
Age (y)^a^	54.5 ± 7.7	53.6 ± 7.8	0.243
Body mass index (kg/m^2^)^a^	23.2 ± 2.8	23.5 ± 2.6	0.163
Red blood cell count (10^4^/μL)^a^	480 ± 37	487 ± 41	0.066
Hematocrit (%)^a^	43.8 ± 3.0	44.3 ± 2.8	0.092
Hemoglobin (g/dL)^a^	15.0 ± 1.2	15.1 ± 1.0	0.199
Mean corpuscular volume (fl)^a^	91.4 ± 4.3	91.2 ± 4.6	0.644
Mean corpuscular hemoglobin (pg)^a^	31.2 ± 1.7	31.1 ± 1.7	0.487
Mean corpuscular hemoglobin concentration (g/dL)^a^	34.2 ± 0.8	34.1 ± 0.8	0.529
White blood cell count (10^2^/μL)^b^	56.8 ± 14.6	60.5 ± 18.3	0.026
Platelet count (10^4^/μL)^a^	22.1 ± 4.8	22.6 ± 5.3	0.322
Coffee consumption (<1 cup per day/1 to 3 cups per day/≥4 cups per day) (%)^c^	44.8/46.2/9.0	36.5/51.2/12.3	0.188
Current smokers (%)^c^	42.1	41.8	0.944
Alcohol consumption (non- or ex-/occasionally/daily) (%)^c^	18.4/35.0/46.6	12.9/40.0/47.1	0.269
Green tea consumption (<1 cup per day/1 to 4 cups per day/≥5 cups per day) (%)^c^	22.6/41.3/36.1	20.0/45.3/34.7	0.689

Age, body mass index, red blood cell counts, hematocrit, hemoglobin, mean corpuscular volume, mean corpuscular hemoglobin, mean corpuscular hemoglobin concentration, white blood cell counts, and platelet counts are given as means ± SD. All *P* values depict significance of differences between Mt5178C and Mt5178A. ^a^Student’s t-test, ^b^Welch’s t-test, ^c^chi-squared test.

Bonferroni correction for multiple comparisons revealed no statistically significant differences in erythrocytic parameters among the three coffee consumption groups (<1 cup per day, 1 to 3 cups per day and ≥4 cups per day) by Mt5178 C/A genotype (Table 2). However, after adjusting for age, BMI, alcohol consumption, habitual smoking and green tea consumption, a significant negative association between coffee consumption and RBC count was seen in participants with Mt5178C (*P* for trend = 0.022). After the aforementioned adjustment, RBC count was significantly lower in those who consumed ≥4 cups of coffee per day than in those who consumed <1 cup of coffee per day (*P* = 0.031). The aforementioned adjustment also revealed a significant negative association between coffee consumption and hemoglobin in participants with Mt5178C (*P* for trend = 0.035). A negative trend between coffee consumption and hematocrit was also observed in participants with Mt5178C, although this did not reach significance (*P* for trend = 0.059). No significant relationships between coffee consumption and erythrocytic parameters were observed in participants with Mt5178A.

**Table 2:** **Erythrocytic parameters by coffee consumption status and Mt5178 C/A genotype**

	Coffee consumption			***P***for trend
	<1 cup per day	1 to 3 cups per day	≥4 cups per day	
**Mt5178C**	N = 119	N = 123	N = 24	
Red blood cell count (10^4^/μL)	480 ± 3	481 ± 3	468 ± 8	0.349
Red blood cell count (10^4^/μL)^a^	484 ± 3	480 ± 3	463 ± 7*	0.022
Hematocrit (%)	43.9 ± 0.3	43.8 ± 0.3	43.1 ± 0.6	0.426
Hematocrit (%)^a^	43.9 ± 0.3	43.5 ± 0.3	42.6 ± 0.6	0.059
Hemoglobin (g/dL)	15.0 ± 0.1	14.9 ± 0.1	14.8 ± 0.2	0.329
Hemoglobin (g/dL)^a^	15.0 ± 0.1	14.8 ± 0.1	14.6 ± 0.2	0.035
Mean corpuscular volume (fl)	91.4 ± 0.4	91.2 ± 0.4	92.2 ± 0.9	0.681
Mean corpuscular volume (fl)^a^	90.8 ± 0.4	90.9 ± 0.4	92.1 ± 0.8	0.275
**Mt5178A**	N = 62	N = 87	N = 21	
Red blood cell count (10^4^/μL)	490 ± 5	485 ± 4	487 ± 9	0.615
Red blood cell count (10^4^/μL)^a^	491 ± 5	483 ± 5	487 ± 9	0.485
Hematocrit (%)	44.2 ± 0.4	44.2 ± 0.3	44.6 ± 0.6	0.668
Hematocrit (%)^a^	44.4 ± 0.4	44.1 ± 0.3	44.4 ± 0.6	0.889
Hemoglobin (g/dL)	15.1 ± 0.1	15.1 ± 0.1	15.2 ± 0.2	0.758
Hemoglobin (g/dL)^a^	15.2 ± 0.1	15.0 ± 0.1	15.1 ± 0.2	0.711
Mean corpuscular volume (fl)	90.6 ± 0.6	91.5 ± 0.5	91.7 ± 1.0	0.247
Mean corpuscular volume (fl)^a^	90.8 ± 0.6	91.5 ± 0.5	91.4 ± 0.9	0.451

^a^Mean ± standard error adjusted for age, body mass index, alcohol consumption, habitual smoking and green tea consumption. Bonferroni correction for multiple comparison was applied. *P <0.05 versus <1 cup of coffee per day.

No significant relationships between coffee consumption and risk of anemia were observed in Mt5178C genotypic men (Table 3). Although not significant, after adjustment for age, BMI, alcohol consumption, habitual smoking and green tea consumption, the risk of anemia may be related to coffee consumption (*P* for trend = 0.078). The adjusted OR for anemia was significantly higher in participants with Mt5178C who consumed ≥4 cups of coffee per day than in those who consumed <1 cup per day (adjusted OR = 3.771, 95% confidence interval: 1.088 to 13.06, *P* = 0.036). In Mt5178A genotypic men, a significant negative association between coffee consumption and the risk of anemia was observed (*P* for trend = 0.049). However, after the aforementioned adjustment, statistical significance disappeared (*P* for trend = 0.137).

**Table 3:** **Odds ratios and 95% confidence intervals for anemia (hemoglobin <14 g/dL) by Mt5178 C/A genotype and coffee consumption**

Genotype and coffee consumption	Frequency (%)		Odds ratio (95% confidence interval)	Adjusted odds ratio ^a^ (95% confidence interval)
	Normal hemoglobin level (hemoglobin ≥14 g/dL)	Anemia (hemoglobin <14 g/dL)		
Mt5178C				
<1 cup per day	99 (83.2)	20 (16.8)	1 (reference)	1 (reference)
1 to 3 cups per day	104 (84.6)	19 (15.4)	0.904 (0.456 to 1.795)	1.140 (0.527 to 2.467)
≥4 cups per day	17 (70.8)	7 (29.2)	2.038 (0.748 to 5.556)	3.771 (1.088 to 13.06)*
			*P* for trend = 0.386	*P* for trend = 0.078
Mt5178A				
<1 cup per day	51 (82.3)	11 (17.7)	1 (reference)	1 (reference)
1 to 3 cups per day	80 (92.0)	7 (8.0)	0.406 (0.148 to 1.115)	0.383 (0.118 to 1.249)
≥4 cups per day	20 (95.2)	1 (4.8)	0.232 (0.028 to 1.915)	0.301 (0.029 to 3.094)
			*P* for trend = 0.049	*P* for trend = 0.137

^a^Odds ratio adjusted for age, body mass index, habitual alcohol consumption, habitual smoking and green tea consumption. *P <0.05.

## Discussion

In the present study, the combined effects of Mt5178 C/A polymorphism and coffee consumption on erythrocytic parameters or the risk of anemia was observed in male Japanese health check-up examinees. An inverse association between coffee consumption and erythrocytic parameters, namely RBC count and hemoglobin, was observed in participants with Mt5178C. The risk of anemia was significantly higher in those who consumed ≥4 cups of coffee per day than in those who consumed <1 cup per day. In participants with Mt5178A, coffee consumption did not appear to influence erythrocytic parameters, but may reduce the risk of anemia.

Although Mt5178C genotype is reported to be overwhelmingly predominant worldwide [29], genetic epidemiological research has reported that the frequency of Mt5178A is 42.1% in the community-dwelling Japanese population [30]. A chi-squared test did not reveal a significant difference between the frequency of Mt5178A in this study and in community-based molecular epidemiological surveys [30], thus suggesting that there is no genetic bias in the participants in this study.

Maternal coffee intake is reportedly a risk factor of both maternal [22, 23] and infant [22] iron deficiency anemia. Coffee consumption is also reported to be one of the risk factors for iron deficiency anemia in preschool children [24]. Fe^55^ and Fe^59^ isotope studies have demonstrated the inhibitory effects of coffee on non-heme iron absorption in human subjects [31]. However, no significant effects of discontinuing coffee consumption on changes in hemoglobin, hematocrit or plasma iron in either non-anemic or anemic children were observed in a randomized intervention study [32]. Moreover, an inverse relationship between coffee consumption and risk of anemia was observed in a large-scale cross-sectional study [33]. Considering these results, the addition of genetic information may contribute to future clinical or epidemiological studies with regard to the effects of coffee consumption on the risk of anemia or iron absorption.

Previous cross-sectional studies reported the joint effects of Mt5178 C/A polymorphism and coffee consumption on the risk of hypertension [16], risk of hyper-LDL cholesterolemia [17] and clustering of cardiovascular risk factors [18]. Amongst men with Mt5178C genotype, the risk of hypertension was significantly lower in those who consumed ≥2 cups of coffee per day than in those who consumed ≤1 cup of coffee per day [16]. The risk of the clustering of cardiovascular risk factors was significantly lower in those who consumed ≥1 cup of coffee per day than in those who consumed <1 cup of coffee per day [18]. Amongst Mt5178A genotypic men, the risk of hyper-LDL cholesterolemia was significantly higher in those who consumed ≥1 cup of coffee per day than in those who consumed <1 cup of coffee per day [17]. Taken together, the present results do not suggest that coffee intake is more beneficial for health in men with Mt5178C than in those with Mt5178A.

Because the success of candidate gene analysis depends on the choice of genes studied, the candidate gene approach alone is generally thought to be insufficient in the study of disease susceptibility or physiological function [34]. Moreover, our research focused on only one mitochondrial DNA polymorphism. Therefore, the biological mechanisms of the combined effects of Mt5178 C/A polymorphism and coffee consumption on erythrocytic parameters remain unknown. However, they probably depend on the biochemical differences in response to some compounds in coffee between ND2-237Leu and ND2-237Met. NADH dehydrogenase is recognized as the major physiological and pathological site of reactive oxygen species (ROS) generation in mitochondria, and itself as a target of assault by ROS [35]. Extrapolation from animal models [36, 37] to humans would suggest that ND2-237Met suppresses ROS production and/or protects NADH dehydrogenase itself from ROS. Coffee intake exerts antioxidant potentials in human subjects [38]. Our previous studies showed that coffee consumption exerts antioxidant behaviors in men with ND2-237Leu rather than in those with ND2-237Met [16–18]. However, it remains uncertain why coffee consumption does not exert antioxidant effects on hematopoiesis in ND2-237Leu genotypic men. Other biochemical effects may play a crucial role in the joint effects of ND2-237Leu/Met polymorphism and coffee consumption on erythrocytic parameters. In any case, elucidating the mechanisms of the joint effects of ND2-237 Leu/Met polymorphism and coffee consumption on erythroid lineage remains a matter for further pathophysiological investigation.

Although the World Health Organization defines the lower limit for normal hemoglobin levels to be 13 g/dL in men [28], many epidemiological studies have adopted hemoglobin levels of 14 g/dL as the lower limit in men [28]. Therefore, anemia was defined as hemoglobin of <14 g/dL in this study. There are many different types of anemia, for example, aplastic anemia, hemolysis, hemorrhage, iron deficiency anemia, sideroblastic anemia and megaloblastic anemia [28]. However, this cross-sectional study did not classify the anemia. Diagnostic approaches to anemia will therefore be required to investigate the joint effects of Mt5178 C/A polymorphism and coffee consumption on the risk of anemia.

As noted above, this study investigated only one mitochondrial DNA polymorphism. Large deletions in mitochondrial DNA are reportedly a hallmark of Pearson’s syndrome, a rare congenital disorder with sideroblastic anemia [27]. Cui *et al*. reported that mitochondrial DNA mutations were associated with aplastic anemia [39]. They found that mutation rates were particularly high in the ND2 coding region in the mitochondrial DNA genomes of patients with aplastic anemia. Mitochondrial genome mutations occasionally result in mitochondrial dysfunctions. Therefore, information on other polymorphisms, mutations or deletions in mitochondrial DNA will be necessary. Moreover, information on causative chromosomal mutations for anemia [28] is also required.

In addition to the lack of anemia classification and information on other mitochondrial DNA genotypes or chromosomal mutations, there are several crucial limitations in this study. First, the study sample was very small. The exploratory nature of study did not allow estimation of sample size in advance. However, post-hoc power analyses for multiple logistic regression analysis, utilizing G*Power 3 [40], indicated that this study was not underpowered. Second, participants comprised only men. Third, we analyzed only a single population; to avoid errors in molecular epidemiological studies, it is necessary to analyze two or more independent data sets. Fourth, this study was a cross-sectional study, and although the study design is able to suggest causal links, it cannot establish valid causality. To overcome these limitations, a prospective cohort study in a larger study sample, including multiple populations, is necessary. Fifth, the evaluation of habitual coffee consumption was based on the number of cups consumed per day. Whether there is any interaction between Mt5178 C/A polymorphism and volume of chlorogenic acids, caffeine or other compounds in coffee on erythrocytic parameters or the risk of anemia warrants further investigation.

## Conclusions

This exploratory cross-sectional study suggests that Mt5178 C/A polymorphism influences the effects of coffee consumption on erythrocytic parameters in male Japanese health check-up examinees. To the best of our knowledge, this is a novel gene-environment interaction on RBC count, hemoglobin and risk of anemia. Although coffee intake is recommended for Mt5178C genotypic men to reduce the risk of atherosclerotic diseases [16, 18], abstaining from coffee intake may be recommended to reduce the risk of anemia. For Mt5178A genotypic men, coffee consumption may prevent anemia. This gene-environment interaction may contribute to both individualized prevention for anemia and cardiovascular diseases, and elucidation of the pathophysiological relationship among mitochondria, coffee consumption and hematopoiesis. Moreover, the study of gene-environment interactions across heterogeneous groups with various lifestyles [1] may provide anthropological insights.

## Abbreviations

BMI: body mass index
Mt5178 C/A: mitochondrial DNA 5178 cytosine/adenosine
LDL: low-density lipoprotein
ND2-237 Leu/Met: NADH dehydrogenase subunit-2 237 leucine/methionine
OR: odds ratio
PCR: polymerase chain reaction
RBC: red blood cell
ROS: reactive oxygen species.
